# Reduction on Proinflammatory Cytokines after Application of Transcutaneous Electrical Nerve Stimulation (TENS) in Patients with a Breast Cancer: A Nonrandomized, Open, and Single-Arm Study Protocol with Paired Analysis

**DOI:** 10.1155/2022/1350813

**Published:** 2022-02-18

**Authors:** Tábata Cristina do Carmo Almeida, Simone Meneghette Zatta, Laércio da Silva Paiva, Luís Eduardo Werneck de Carvalho, Jean Schoueri, Luiz Carlos de Abreu, Fernando Luiz Affonso Fonseca, Fernando Adami

**Affiliations:** ^1^Centro Universitário FMABC, Laboratório de Epidemiologia e Análise de dados, Av Lauro Gomes, 2000, Vila Sacadura Cabral, Santo André, São Paulo (SP), CEP: 09060-870, Brazil; ^2^Pesquisare Saúde, Clinical Research, Av Dom Pedro II, 125, Room 53, Jardim, Santo André, São Paulo 09060-110, Brazil; ^3^Oncológica do Brasil Ensino e Pesquisa, Av. Visc. de Souza Franco, 570, Reduto, Belém, Pará 66053-000, Brazil; ^4^Centro Universitário FMABC, Laboratório de Delineamento de Estudos e Escrita Científica, Av. Lauro Gomes, 2000, Vila Sacadura Cabral, Santo André, São Paulo (SP), CEP: 09060-870, Brazil; ^5^Centro Universitário FMABC, Laboratório de Análise Clínicas, Av. Lauro Gomes, 2000, Vila Sacadura Cabral, Santo André, São Paulo (SP), CEP: 09060-870, Brazil; ^6^Universidade Federal de São Paulo (UNIFESP), Departamento de Ciências Farmacêuticas, Diadema, São Paulo (SP), Brazil

## Abstract

**Background:**

Transcutaneous electrical nerve stimulation (TENS) has been used as analgesic therapy in many diseases. It is already known that studies that have observed the relationship between pain and cytokines have found that patients who report less severe pain have less production of proinflammatory cytokines. However, one another accepted mechanism is that decreasing proinflammatory cytokines results in decreased pain intensity. Analyzing the literature, the authors describe that, in addition to the analgesic effect, TENS has shown systemic effects, and clinically, the reduction of proinflammatory cytokines could be a protective factor against inflammation. To test the inflammatory effect of TENS, we researched the literature for clinical conditions that suggest that proinflammatory cytokines are one of the main mediators of the disease process. Chronic inflammation is one of the risk factors mentioned for the development of a new cancer; at the same time, it is indicated as an indicator of the worst prognosis. Studies also suggest that the worst prognosis of breast cancer, one of the types with the highest incidence in the world, may be related to increased inflammatory activity. Considering that inflammation is increased in breast cancer and that TENS can reduce proinflammatory cytokines even without blocking the pain pathway, our hypothesis is that the anti-inflammatory effect of TENS can bring benefits to these patients. The aim of this study will be to evaluate the effect of TENS on blood reduction of proinflammatory cytokines in breast cancer patients.

**Methods:**

This study will evaluate at least 59 patients, over 18 years of age, diagnosed with breast cancer, but who have not yet started any treatment. All patients will be submitted to TENS intervention (Ibramed, Model Neurodyn III, parameters: VIF—turn on, frequency—2-247 Hz, pulse size—50-500 *μ*s, and intensity (mA)—maximum tolerated by the patient), and the data will be analyzed in the pre- and postintervention of each patient. The application has a total duration of 30 minutes, and 8 ml of blood will be collected before and after the intervention. Proinflammatory (IL-1, IL-2, IL-6, IL-7, and TNF-*α*) and anti-inflammatory (IL-4, IL-10, IL-13, and FTC*β*) cytokines will be analyzed. As a primary endpoint, we will analyze the reduction in blood concentration of proinflammatory cytokines, and as secondary endpoints, we will analyze the size of the effect according to each type of proinflammatory cytokine, describe the effect size of the reduction according to the breast cancer immunohistochemistry, and analyze the effect of TENS on anti-inflammatory cytokines. This study is approved by the Research Ethics Committee (Centro Universitário FMABC, Brazil) and registered in the Brazilian Clinical Trials (Search text: RBR-10jbwh47).

## 1. Background

Used since 1970 as an adjunct therapy to control acute and chronic pain in a lot of medical and surgical conditions, transcutaneous electrical nerve stimulation (TENS) is a noninvasive physiotherapeutic resource that acts to block pain through the central nervous system [1–5].

After pain is present, there are two mechanisms of action that may be involved: (i) blocking nociception reduces the production of proinflammatory cytokines or (ii) decreasing proinflammatory cytokines that decrease pain intensity [6–8].

It is already known that studies that have observed the relationship between pain and cytokines have found that patients who report less intense pain have less production of proinflammatory cytokines [9, 10].

The mechanism block pain described above was thought of for the hypothesis that the TENS, a physiotherapeutic resource that acts in the path of pain, could reduce cytokines. To answer the question, a systematic review of randomized clinical trials was carried out, and it was found in the meta-analysis that TENS significantly reduced blood proinflammatory cytokines compared to the control group [11].

Analyzing the literature, the authors describe that in addition to the analgesic effect, the electric current has also shown systemic effects, and one of those that is mentioned is the anti-inflammatory effect [6, 12–15].

In the systematic review, we observed that one article evaluated the reduction of cytokines and pain [12]. The other articles evaluated the reduction of cytokines in chronic diseases such as COPD [16] and rheumatoid arthritis [16, 17] and also during surgery [18].

In the research of Wang et al. [18], for example, which evaluated outcomes during a brain surgery, the use of TENS showed a reduction in intraoperative cytokines while the control group had an increase. Considering that inhibiting the inflammatory response in the brain (reduction of proinflammatory cytokines) can minimize brain damage, treatments with TENS may improve the prognosis after surgery.

By analyzing the studies related to chronic disease [16–19], it was hypothesized that TENS could act firstly in the cytokine reduction pathway. If TENS has this effect even without pain being present, clinically the reduction of proinflammatory cytokines could be a protective factor against inflammation.

For testing the effect of TENS on cytokines without block pain, we searched the literature for clinical conditions suggesting that the proinflammatory cytokines are one of the main mediators of the disease process.

The chronic inflammatory process can be related to an increased risk to develop cancer, since it implies changes in the tissue microenvironment [20, 21]. This tissue change induces cell proliferation, promotes angiogenesis, and increases the survival of tumor cells (because it inhibits their apoptosis). That influences the migratory behavior and contributes to cellular dissemination and metastasis [22–24].

Whenever inflammatory cells secrete a variety of factors, including chemokines and cytokines [25], they further promote tumor progression instead of promoting an antitumor response [26].

The cancer immune system is related to the production of catabolic signals and stimulation of the production of proinflammatory cytokines and growth factors by tumor cells. The increase of Tumor necrosis factor alpha (TNF-α) paired increase of interleukins might be related a worst prognosis in cancer. The chronic inflammation can result in an increased possibility of developing a new cancer [27].

Several types of cancer are studied in the literature, but the relationship between chronic inflammation and cytokines was initially observed in breast cancer [20, 21, 28]. The studies suggest that the worst prognosis of breast cancer may be related to increased inflammatory activity [20, 21, 28].

Breast cancer is one of the types with the highest incidence in the world, and because of that, several topics are developed to identify potential risk factors and protection against cancer [29–31].

Considering that inflammation is increased in breast cancer and that TENS can reduce proinflammatory cytokines even without blocking the pain pathway, our hypothesis is that the anti-inflammatory effect of TENS can cause benefit for these patients. The aim of this study will be to evaluate the effect of TENS on blood reduction of proinflammatory cytokines in patients with breast cancer.

## 2. Study Rationale

TENS is a noninvasive, easy-to-apply, and low-cost therapeutic resource. Several studies have pointed out the benefits of TENS in a variety of pathologies, and, so far, no deleterious effects of the use of TENS are known.

The main clinical benefit would be that the reduction of proinflammatory cytokines (protective factor against inflammation) could be applied in diseases that have inflammatory causes or inflammatory complications. We could use TENS as a regulator of the inflammatory process.

Regarding the research risks, we can mention complications resulting from procedures such as skin irritations and a small cutaneous hematoma during blood collection.

## 3. Study Objectives and Study Design

A protocol of clinical trials was developed following the guidelines of Standard Protocol Items: Recommendations for Interventional Trials (SPIRIT) [32]. The checklist for SPIRIT can be seen in Supplementary Material 1.

**Figure 1:**
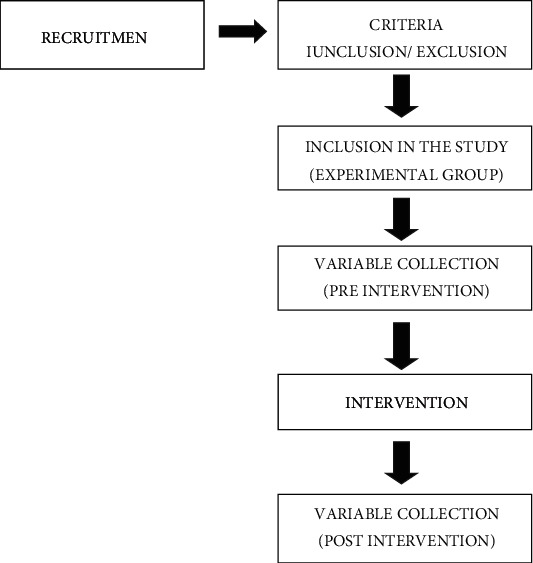
Trial flow diagram.

The objective of this study is carried out to assess the reduction of proinflammatory cytokines after TENS application in patients with breast cancer. A nonrandomized, open, single-arm clinical trial with paired analysis will be developed. The study design is described in Figure 1.

A minimum of 59 patients is expected. All subjects will receive a single application of TENS for 30 minutes. Assessments will be made before and after application. Screening data will be reviewed to determine subject eligibility. Individuals who meet all of the inclusion criteria and none of the exclusion criteria will be included in the study. The total duration of the subject's participation will be one day (time to complete all study evaluations).

## 4. Criteria for Evaluation

The endpoints for the study are described below:

*Primary Endpoint*. The primary endpoint is to analyze the reduction in blood concentration of proinflammatory cytokines.

*Secondary Endpoint*. The secondary endpoints are to analyze the effect size according to each type of proinflammatory cytokine, describe the effect size of the reduction according to breast cancer immunohistochemistry, and analyze the effect on anti-inflammatory cytokines.

## 5. Subject Selection and Recruitment Strategies

Women diagnosed with breast cancer who meet the inclusion and exclusion criteria will be eligible to participate in this study. The eligibility criteria are described in Table 1.

The study will be carried out on the premises of the clinical research site (Pesquisare Saúde, Santo André, Brazil), and these patients will be recruited as they arrive at the site to be evaluated in the protocols.

Patients are referred to the research site by doctors and partner hospitals who have already been informed of ongoing breast cancer studies. The research criteria have already been passed to all partners, and as soon as a patient is identified, they are invited to go to the research site where all the information is explained in more detail.

## 6. Study Intervention

The consent form will be obtained as the first procedure from all participants by the study coordinator. The confidentiality of the data will be guaranteed until the end of the research.

The study will not be randomized. All patients will be submitted to TENS intervention, and the data will be analyzed in the pre- and postintervention of each patient.

### 6.1. Blinding

Since it is a sensory current, patients will be blinded regarding the modulation of the current (TENS programming values), but not for the sensation and intensity that must be perceived during the application. All of the patients will be asked about the tingling perception that the current causes, and the intensity limit will be the maximum sensation that is tolerated.

The laboratory technicians will be blinded about the study design and will receive the samples identified by number and the indication of pre and post.

### 6.2. Intervention

A single application will be performed for only 30 minutes on each patient.

**Figure 2:**
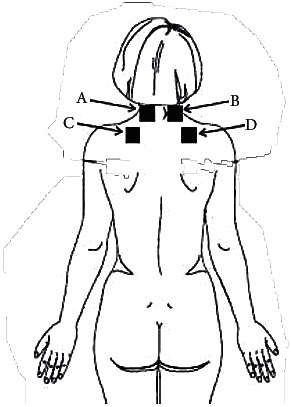
Demonstration of the TENS application site.

The area chosen for application will not expose parts of the body and will be easy to apply. To standardize the application, anatomical points will be used as a reference. The location of the placement of the self-adhesive electrodes (5 cm) will be shown in Figure 2. The upper points (electrodes A and B) are three fingers above the spinous process of C7, while the lower points (electrodes C and D) are above the spine of the scapula, above the tender point corresponding to the trapezius muscle.

TENS equipment (Ibramed, Model Neurodyn III, Amethyst Line, two channels) will be used during the study.

The parameters of application of TENS can be adjusted in several ways, but studies have already shown that patients tend to show sensitivity accommodation with a continuous stimulus. Studies that evaluated the decrease in pain compared to control groups obtained better results using the TENS modality with variable intensity and frequency (VIF). These results suggest that it is important to adjust the pulse amplitude during the application of TENS to obtain maximum and longer-lasting analgesic effects when compared to fixed parameter modalities [33, 34].

Regarding the intensity adjustment, a study that evaluated dose response in relation to pain showed that stronger intensities had greater effect. For this reason, it is suggested that the intensity of TENS be applied at the highest possible intensity to obtain maximum pain relief [35].

For the application of the TENS current, the programming is stipulated as described in Table 2.

## 7. Study Procedures (Day 1)

All procedures will be performed within the premises of Pesquisare Saúde (Santo André, Brazil), in the space already equipped and suitable for carrying out international clinical research, and the analysis of blood will be made by the Clinical Analysis Laboratory of the Centro Universitário FMABC (Brazil).

On the day of treatment (D1), after signing the informed consent form (ICF), women will complete an evaluation questionnaire with sociodemographic information, medical history, behavioral habit history, and history of breast cancer (e.g., breast cancer diagnosis, symptoms, staging, and indication for chemotherapy). The questionnaire is described in Supplementary Material 2.

### 7.1. Clinical Laboratory Measurements

The blood will be collected and sent to the clinical laboratory. The study's dependent variables will be the proinflammatory cytokines interleukin 7 (IL-7), interleukin 6 (IL-6), interleukin 2 (IL-2), interleukin 1 (IL-1b and IL-1*α*), and TNF alpha (TNF-*α*) and the anti-inflammatory cytokines interleukin 4 (IL-4), interleukin 4 (IL-10), interleukin 13 (IL-13), and transforming growth factor *β* (FTC*β*).

### 7.2. Data Collection and Analysis Methods

The electrodes for applying TENS will be positioned as described previously (Figure 2). After placement, the patient will be accommodated in an armchair where she will remain until the end of the intervention. 8 ml of blood divided into 4 tubes (2 purple tubes of EDTA and 2 yellow tubes of biochemistry) will be collected by peripheral puncture, in the patient's chosen arm. The tubes will be identified as precollection. In order to make the process less invasive, the patient will have venous access until the end of the second collection.

After the end of the 30 minutes of TENS, the same collection process described above will be repeated. The vials will be identified as postcollection.

Blood collection will take place where the TENS intervention will be carried out (Pesquisare Saúde, Santo André). After collection, the samples will be sent on the same day, at room temperature, to the Clinical Analysis Laboratory of the Centro Universitário FMABC (Santo André, Brazil).

Quantitative analysis of serum levels of proinflammatory cytokines will be performed using the enzyme-linked immunosorbent assay (ELISA) technique (BD OptEIA, Biosciences), following the manufacturer's guidelines and adopting the instructions in the manual as a reference value.

## 8. Statistical Methods

### 8.1. Sample Size

The sample size was based on the calculation of the sample size in pilot studies proposed by Viechtbauer et al. [36]: 5% probability with 95% confidence level resulted in a sample requiring 59 participants.

### 8.2. Statistical Analysis

For the characterization of the sample, a descriptive analysis of the data will be performed; therefore, the qualitative variables will be described by absolute and relative frequency; the quantitative variables that present normal distribution according to the Shapiro-Wilk test (*p* ≥ 0.05) will be presented as means, standard deviations, and 95% confidence intervals, while those that do not present normal distribution according to the Shapiro-Wilk test (*p* < 0.05) will be presented as medians and respective 95% confidence intervals and 25th and 75% percentiles.

To analyze the quantitative variables between the groups, Student's *t*-test will be used for variables that have a normal distribution and the Mann-Whitney test will be used for variables that do not have a normal distribution.

The confidence level adopted for all analyses will be 95%. All analyses will be performed using the Data Analysis and Statistical Software for Professionals (Stata) version 11.0®.

## 9. Ethical and Regulatory Considerations

The study was approved by the Independent Ethics Committee (Centro Universitário FMABC, Brazil) and is also registered in the Brazilian Clinical Trials (Search text: RBR-10jbwh47).

Ethical considerations will be based on the use of biological material for scientific purposes, with confidentiality of identity, free from coercion or conflict of interest of the institution or of people involved in the project.

The collections will respect the technical protocols of the services involved. Research participants will be previously informed of the therapy by TENS and blood collection. The analysis of these data will only happen under the consent in a specific form (Informed Consent Form (ICF)), according to resolution 466/2012 of the National Health Council. The present project has been already approved by the Ethics Research Committee.

## 10. Discussion

TENS acts on afferent nerve fibers blocking the nerve transmission of pain—an effect known as gate theory [37]—or stimulating the release of opioids through the central nervous system [12, 37]—both action mechanisms are already described to analgesia.

Regarding studies with TENS and pain reduction [1–5], the results evidence that there is a reduction in pain intensity when compared to control groups in a variety of diseases. When evaluating studies related to pain intensity, it is observed that less intense pain also has fewer cytokines [7, 9, 10]. The action mechanism through blocking pain (gate theory) may induce the reduction in cytokines.

In order to test this hypothesis of the pain blockade (TENS)/cytokine pathway, we developed a systematic review with meta-analysis [11]. The effect of reducing cytokines was statistically significant, but in detriment of the few researches, it was unable to determine the consistency of this effect and the action mechanism involved in this result. Due to the heterogeneity of the clinical trials found, we cannot rule out that the results could be related to another TENS action pathway (opioid release by the central nervous system)

The researches indicate that acupuncture or electroacupuncture facilitates the release of certain neurotransmitters, especially opioids [38]. The same opioid release is described as an action mechanism of TENS current by electrodes [37, 39]. These opioids act on the central nervous system and activate the sympathetic or parasympathetic nervous system [37, 38].

As opioid receptors are also present on immune cells, TENS-induced opioid release could directly modulate these cells (neuroimmune interaction) [38]. The activation of the parasympathetic nervous system releases acetylcholine (the main neurotransmitter), which by stimulating macrophages can inhibit the production of several proinflammatory cytokines TNF-*α*, IL-1, and IL-6 [6, 40].

The systemic inhibition of immunity via parasympathetic flow is the path that would explain the anti-inflammatory effect of several nonpharmaceutical modalities (e.g., acupuncture, biofeedback, and placebo effect) [41]. We hypothesized that TENS works by systemic inhibition and that cholinergic interactions would lead to the reduction of proinflammatory cytokines that we found in our systematic review. And in order to test this hypothesis, we chose a disease mediated by cytokines but without the pain bias.

Recent research indicates that the neoplasic environment arises from sites of infection, chronic irritation, and inflammation and that inflammation is a component of tumor progression. Coussens and Werb, cited by Kavoussi and Ross [41], report that the tumor microenvironment is largely influenced by inflammatory cells, and this process would be responsible for proliferation, survival, migration, and metastasis.

The primary objective of this protocol is to assess whether TENS can reduce proinflammatory cytokines in an inflammatory disease. Cancer was chosen as the study pathology, considering that studies describe that the chronic inflammation present in the tumor microenvironment increases the risk of developing or disseminating cancer [20–23]. Among the types of cancer that could be studied, we chose breast cancer because it is one of the carcinomas with higher incidence and has the worst prognosis when related to increased inflammatory activities [20, 21, 28–31].

Our hypothesis is that if we have a successful conclusion of the proposed clinical trial, we will identify this anti-inflammatory effect on TENS possibly related to a parasympathetic nerve pathway. We pretend to explore the results and calculate the sample size for other clinical trials that can test and increase the power of evidence of TENS (anti-inflammatory effect) in the treatment of inflammatory diseases.

## Figures and Tables

**Table 1:** Eligibility criteria.

Inclusion criteria
(i) Written informed consent obtained from the subject (ii) Adult women (≥18 years of age) (iii) Primary diagnosis of breast cancer confirmed by immunohistochemistry and breast biopsy (iv) Medical indication but having not started chemotherapy
Exclusion criteria
(i) Metastatic disease, breast cancer recurrence, or mastectomy (ii) Bilateral breast cancer (iii) Chemotherapy, biological therapy, radiation therapy, or previous surgery for any active malignancy, including breast cancer (iv) No biological therapy even if indicated for another pathology (v) Another malignancy in the last 5 years (vi) Any pathology in the cervical spine and shoulders, even if asymptomatic (vii) Any inflammatory disease and/or orthopedic disease in activity (viii) Physical therapy or acupuncture sessions ongoing for any pathology (ix) Heart failure (New York Heart Association) grade III or IV, myocardial infarction, coronary/peripheral artery revascularization, stroke, unstable angina pectoris, uncontrolled arrhythmia, or pulmonary embolism (x) Active infection (xi) Uncontrolled diabetes mellitus (xii) Uncontrolled hypertension (blood pressure > 150/100 mmHg) (xiii) Major surgery or significant traumatic injury lesser than 6 months

**Table 2:** TENS parameters.

Current	TENS
VIF (variable intensity and frequency)	Turn on
Frequency—R (Hz)	2-247 Hz
Pulse size—T (*μ*s)	50-500 *μ*s
Intensity (mA)	Maximum tolerated by the patient
Time	30 minutes

## Data Availability

The data used to support the findings of this study may be available upon request to the authors, who can be contacted by e-mail.

## Abbreviations

CA: Cancer
D1: Day 1 of treatment
ES: Effect size
EDTA: Ethylenediaminetetraacetic acid
ELISA: Enzyme-linked immunosorbent assay
Hz: Hertz
IL-1*β*: Interleukin 1*β*
IL-1*α*: Interleukin 1*α*
IL-2: Interleukin 2
IL-4: Interleukin 4
IL-6: Interleukin 6
IL-7: Interleukin 7
IL-10: Interleukin 10
IL-13: Interleukin 13
*μ*s: Microsecond
mA: Milliampere
SPIRIT: Standard Protocol Items: Recommendations for Interventional Trials
TENS: Transcutaneous electrical nerve stimulation
TGF-*β*: Transforming growth factor *β*
TNF-*α*: Tumor necrosis factor alpha
VIF: Variable intensity and frequency
WIC: Written informed consent.

## Supplementary Materials

Supplementary 1 Supplementary Material 1: SPIRIT checklist.

Supplementary 2 Supplementary Material 2: evaluation questionnaire.
