# Supplementary material for: Reduction on Proinflammatory Cytokines after Application of Transcutaneous Electrical Nerve Stimulation (TENS) in Patients with a Breast Cancer: A Nonrandomized, Open, and Single-Arm Study Protocol with Paired Analysis
Source: Mediators Inflamm. 2022 Feb 18;2022:1350813. doi: 10.1155/2022/1350813 (PMC8886802; doi:10.1155/2022/1350813)
Supplement: Supplementary 2 — Supplementary Material 2: evaluation questionnaire. [file 1350813.f2.docx]

**SUPPEMENTARY MATERIAL 2: QUESTIONNAIRE**

PATIENT: __________________________________________ ID:________________

BIRTHDATE _____/______/_______ AGE: __________

WEIGHT: __________________ HEIGHT: _____________ RACE: ______________

**1. MEDICAL HISTORY**

| **MEDICAL HISTORY** | | **DATE START** | **DATE END** |
| --- | --- | --- | --- |
| Chronic heart disease (heart attack, stroke, angina) | YES  NO |  |  |
| Hypertension | YES  NO |  |  |
| Chronic Pulmonary disease/ Asthma | YES  NO |  |  |
| Vascular disease | YES  NO |  |  |
| Chronic kidney disease | YES  NO |  |  |
| Chronic liver disease | YES  NO |  |  |
| Colitis / Crohn's Disease | YES  NO |  |  |
| Diabetes | YES  NO |  |  |
| Obesity | YES  NO |  |  |
| Dyslipidemia | YES  NO |  |  |
| Another malignant neoplasm | YES  NO |  |  |
| Chronic hematological disease | YES  NO |  |  |
| Autoimmune disease (Lupus, multiple sclerosis, celiac disease, etc.) | YES  NO |  |  |
| Ankylosing spondylitis | YES  NO |  |  |
| Osteoarthritis | YES  NO |  |  |
| Psoriatic arthritis | YES  NO |  |  |
| Rheumatoid arthritis | YES  NO |  |  |
| Dermatitis | YES  NO |  |  |
| Anxiety | YES  NO |  |  |
| Depression | YES  NO |  |  |
| Psoriasis | YES  NO |  |  |
| Orthopedic disease | YES  NO |  |  |
| Other | YES  NO |  |  |

| **2. SURGERY HISTORY**  Does the patient have any surgical history?  ( ) YES ***^If YES, insert the surgery below^** ( ) NO  ^1 ______________________________________________________________________________________________________ date _____/________/_________^  ^2 ______________________________________________________________________________________________________ date _____/________/_________^  ^3 ______________________________________________________________________________________________________ date _____/________/_________^  ^4______________________________________________________________________________________________________ date _____/________/_________^  ^5 ______________________________________________________________________________________________________ date _____/________/_________^ |
| --- |

| ***3. HISTORY OF USE OF SUBSTANCES*** | |
| --- | --- |
| 1. *ALCOHOL* | ( ) None  ( ) Moderate  ( ) Intense |
| 2. *TABAGISM* | ( ) Never  ( ) Current Start: __________ Nº cigarrettes______  ( ) Former Start: __________ Nº cigarrettes______  End: _____________ |
| 3. *SPORTS* | ( ) NO  ( ) YES ________________________________ |

| **4. BREAST CANCER HISTORY** | |
| --- | --- |
| *1. Diagnostic* | ( ) Biopsy _____________________________  ( ) Immunohistochemistry ____________________ |
| *2. Chemotherapy indication* |  |
| *3. Breast Cancer Stages* | **Histologic**  ( )GX ( )G1 ( )G2 ( )G3  **Primary tumor (T)**  ( ) TX ( )T 0 ( )Tis ( )T1 ( )T2 ( )T3 ( )T4  **Regional Limph Nodes (N)**  ( )NX ( )N0 ( )N1 ( )N2  **Distant Metastases (M)**  ( ) MX ( )M0 ( )M1  **Stage Grouping**  ( )Stage 0 ( ) Stage I A ( ) Stage II B  ( ) Stage IIA ( ) Stage IIB  ( ) Stage IIIA ( )Stage IIIB ( )Stage IIIC  ( ) Stage IVA  HER 2 ( ) + ( ) –  ER ( ) + ( ) –  PR ( ) + ( ) – |
| *3. Date diagnostic* |  |
| *4. Symptoms* |  |

**CHECK LIST ELEGIBILITY CRITERIA**

| **INCLUSION** | |
| --- | --- |
|  | - Adult women (≥18 years of age); |
|  | - Primary diagnosis of breast cancer confirmed by immunohistochemistry and breast biopsy; |
|  | - Indication, but not having started chemotherapy |
| **EXCLUSION** | |
|  | - Metastatic disease, breast cancer recurrence or mastectomy; |
|  | - Bilateral breast cancer. |
|  | - Chemotherapy, biological therapy, radiation therapy or previous surgery for any active malignancy, including breast cancer. |
|  | - No biological therapy even if indicated for another pathology. |
|  | - Another malignancy in the last 5 years. |
|  | - Any pathology in the cervical spine and shoulders, even if asymptomatic. |
|  | - Any inflammatory and / or orthopedic disease; |
|  | - Be undergoing physical therapy or acupuncture sessions for any pathology. |
|  | - Heart failure (New York Heart Association) III or IV; myocardial infarction, coronary / peripheral artery revascularization, congestive heart failure, stroke, unstable angina pectoris, uncontrolled arrhythmia or pulmonary embolism |
|  | - Active infection. |
|  | - Uncontrolled diabetes mellitus. |
|  | - Uncontrolled hypertension (blood pressure> 150/100 mmHg) |
